# Usability study of pH strips for nasogastric tube placement

**DOI:** 10.1371/journal.pone.0189013

**Published:** 2017-11-30

**Authors:** Simone Borsci, Peter Buckle, Jeremy Huddy, Zenia Alaestante, Zhifang Ni, George B. Hanna

**Affiliations:** National Institute for Health Research Diagnostic Evidence Cooperative of London, Faculty of Medicine, Department of Surgery & Cancer, Imperial College, London, United Kingdom; Universita degli Studi di Perugia, ITALY

## Abstract

**Aims:**

(1) To model the process of use and usability of pH strips (2) to identify, through simulation studies, the likelihood of misreading pH strips, and to assess professional’s acceptance, trust and perceived usability of pH strips.

**Methods:**

This study was undertaken in four phases and used a mixed method approach (an audit, a semi-structured interview, a survey and simulation study). The three months audit was of 24 patients, the semi-structured interview was performed with 19 health professionals and informed the process of use of pH strips. A survey of 134 professionals and novices explored the likelihood of misinterpreting pH strips. Standardised questionnaires were used to assess professionals perceived usability, trust and acceptance of pH strip use in a simulated study.

**Results:**

The audit found that in 45.7% of the cases aspiration could not be achieved, and that 54% of the NG-tube insertions required x-ray confirmation. None of those interviewed had received formal training on pH strips use. In the simulated study, participants made up to 11.15% errors in reading the strips with important implications for decision making regarding NG tube placement. No difference was identified between professionals and novices in their likelihood of misinterpreting the pH value of the strips. Whilst the overall experience of usage is poor (47.3%), health professionals gave a positive level of trust in both the interview (62.6%) and the survey (68.7%) and acceptance (interview group 65.1%, survey group 74.7%). They also reported anxiety in the use of strips (interview group 29.7%, survey group 49.7%).

**Conclusions:**

Significant errors occur when using pH strips in a simulated study. Manufacturers should consider developing new pH strips, specifically designed for bedside use, that are more usable and less likely to be misread.

## Introduction

The use of pH strips is critical to clinical decision when using NG-tube for feeding. NG-tubes are inserted into the stomach via the nose to feed patients. Only pH testing and chest radiography are reliable and currently recommended [1] approaches to ascertain NG-tube placement. In the USA, most procedures rely only on the use of x-ray to confirm the tube position before feeding, while European countries often apply a combination of pH strips and an x-ray to achieve the same goal.

The use of x-ray allows clinicians to visually confirm the tube position. This decreases the uncertainty and thus provides a high level of confidence that the tube is in the stomach. Nevertheless, radio opacity of tubes is not always satisfactory [2], and more than 50% of tubes could either migrate from the stomach or be inadvertently removed by patients [2–4] after the x-ray has been taken. These factors create uncertainty that can lead to repeat chest radiography, exposing patients to multiple radiation exposures and may delay the administration of feeding or medications[5]. In tune with that, especially in Europe, often an x-ray is seen as a second line of checking the tube placement which can be used when the pH approach has failed [6]. Although, the use of pH strips or x-ray (or a combination of these) is standard practice to confirm NG-tube placements, errors can occur during the confirmation process. This may expose patients to high risks when the tube is in their lungs[6] instead of in their stomach.

There also appears to be an assumption that pH strips, being a low-cost strip of paper to detect pH of aspirated gastric contents, can be readily and reliably used by healthcare staff. This is seen to be a simpler, quicker and cheaper process that the use of x-ray to confirm tube placement. It is also seen as faster thus speeding up the feeding of patients [7]. Clinicians are aware that limitations of pH and X-ray processes can lead both to a risk of NG-tube misplacement and delays in feeding patients [7–9]. Despite this, we could find no published literature that has considered their usage and their usability by health professionals when pH strips are used to ascertain the position of the NG-tubes. In fact, the literature focuses on advantages and disadvantages of both the approaches to identify and eliminate inaccurate NG-tube position tests [1, 5, 10] and shows that false negative results with pH strips can occur when patients receive acid inhibitors[1, 5, 8]. Whilst some authors admit that human errors in reading pH strips could affect patient safety [8], we identified only one conference paper [11] in which researchers discussed the likelihood of misreading the pH strip in clinical procedures. In this simulated setting, a small group of 10 nurses were tested using pH strips impregnated by predetermined pH solutions. Nurses incorrectly read about 30% of pH measurements, and 12% of pH 6.0 readings were misinterpreted as having a pH of 5.5. These are critical values in determining whether a tube is or is not within the stomach.

The lack of a field study on the misreading of pH strips could be due to the fact that it is currently impossible to verify, retrospectively, the reading of pH strips. After use, strips are disposed of and only the pH levels are reported in patient notes. Shadowing clinicians or asking them to store pH strips for a second reading is not feasible because the validity of the reading could be established only by knowing the pH of the gastric contents.

This study aimed to estimate the likelihood of errors in decision making using pH strips, and to investigate the reasons behind any potential errors. Our investigation sought to answer the following four questions:

1. Is the use pH strips perceived as simple and safe by clinicians?
2. What is the likelihood of misreading the pH paper that could affect clinicians’ decision making?
3. What are the causes of errors?
4. What are the potential mitigation strategies?

To answer these questions the study examined i) the current clinical use of pH strips through a human factor analysis of the health professional’s acceptance, trust and perceived usability of pH strips; ii) tested (through simulation) the likelihood that a pH strip test is misinterpreted; and iii) involved clinicians in interviews to understand the context of use, identify the cause of errors and determine potential mitigation strategies.

## Methods

**Fig 1:**
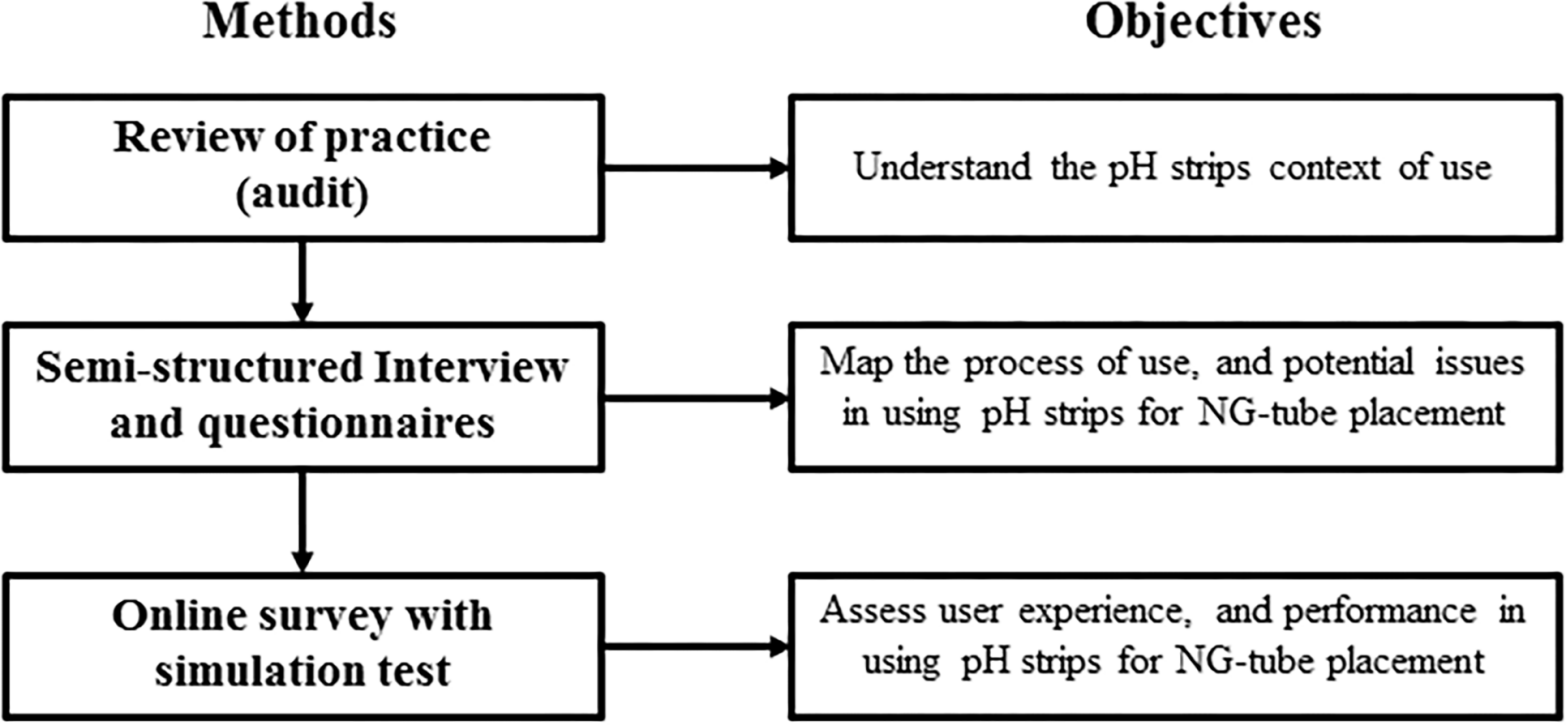
Summary of methods and objectives of the study.

A human factors approach was taken to answer our questions by exploring the use of pH strips throughout a review of practice, a semi-structured interview and an online survey with a simulation test to model the ability of participants to correctly read pH levels reported by strips (Fig 1). Approval for clinical audit was provided by the Clinical Governance committee (CG approval REF: SCC_GEN_1516_002) and approval for service evaluation was provided by the Service Evaluation committee (SE approval REF: SE127). Both of these committees reviewed the research protocol which described the study methods and included information regarding how participants’ informed consent was obtained for different parts of the study (including for the clinician interviews and online survey/simulation–for which written informed consent was obtained in both cases). The approved protocols also provided details regarding the audit, including the accessing of patients’ medical records. No written consent was obtained for accessing medical records because there was not patient contact or involvement, and patient data were anonymised.

IBM^®^SPSS^®^ statistics version 22.0 was used to perform descriptive and inferential statistics, and reliability analysis.

### Review of practice

An audit of current practice for the confirmation of current NG tube placement was undertaken in a large London NHS acute hospital trust (by two of the authors ZA and JH). The audit was limited to general surgical, ear, nose and throat and stroke wards due to their increased use of NG tubes. The audit standard was the trust guidelines for NG tube insertion. Data were gathered from patient medical and nursing notes regarding the number of procedures per patient, number of cases in which aspiration cannot be achieved, the needs of x-ray, and time to start the feeding or medication after pH or x-ray confirmation. All the procedures were carried out by using the same type of hydrophilic NG-tube, and CE marked strips. Data collection took place from February to April 2016.

### Semi-structured interview

An initial literature review [6, 12, 13], and task analysis [14] led by a human factors expert (SB) and a nurse (ZA) was used to build a preliminary graphic representation of the tasks involved in the use of NG-tube and pH strips (S1 Fig). This graphic was developed in line with the UK National Patient Safety Agency Guidelines [6, 12] by using the software TaskArchitect v.3.1. A user focused approach [15, 16] was then used with a group of five professionals, including nurses and clinicians, to revise the graphic representation of NG-tube and pH strips use. Professionals were invited to add/remove or amend steps in the procedure, to explain each step and to comment on issues they had encountered. They were also asked to describe the strategies they had used to solve problems and enable them to complete the task. The outcome of this process was a revised graphic presentation (S2 Fig). This was used at the beginning of the interview to involve participants in a co-design session and to help them to focus on the topic of the study.

The interviews, reported here are in keeping with consolidated criteria for reporting qualitative research guidelines [17], took place from March to June 2016. Professionals were invited by email, convenience sampling was undertaken, and potential participants were included based on their declared experience with the procedure. The face-to-face interviews were undertaken by one author (SB) who is a male, human factors expert at Imperial College.

The interviewer did not know any of the participants before the investigation. The participants received information on the interviewer, background and aims of the research project before the interview. Transcripts were not returned to participants unless clarification was required and no repeat interviews were undertaken. Interviews were analysed by two of the authors (SB and PB) who independently reviewed the transcripts using constant comparative techniques before meeting to compare emergent themes. Insights and themes emerged from the interview were used to compose the final version of the graphic model of NG-tube and pH strips use. Interviews were undertaken until saturation had been reached, as demonstrated by the absence of new themes emerging from analysis.

As part of the interview, participants were also invited to complete a demographic and qualitative questionnaire about their experiences in the use of pH strips in the last 12 months (S1 Appendix). Moreover, standardised questionnaires were used to assess three factors namely (S2 Appendix):

- **Perceived user experience and usability** of pH strips was evaluated by the LITE version of the Usability Metric for User Experience (UMUX-LITE) [18–20]. Through an adjusted formula [19], UMUX-LITE results are compared to the standardised grading scale proposed by Sauro and Lewis [21]. This provides a standard usability grade to a tool in a scale from grade F (lowest) to A++ (highest);
- **Acceptance** of pH strips use was measured through an adapted Technology Acceptance Model questionnaire (TAM) [22];
- **Professional trust** in pH strips was assessed through an adapted Trust In Use scale (TIU) [23].

### Online survey and simulation test

**Fig 2:**
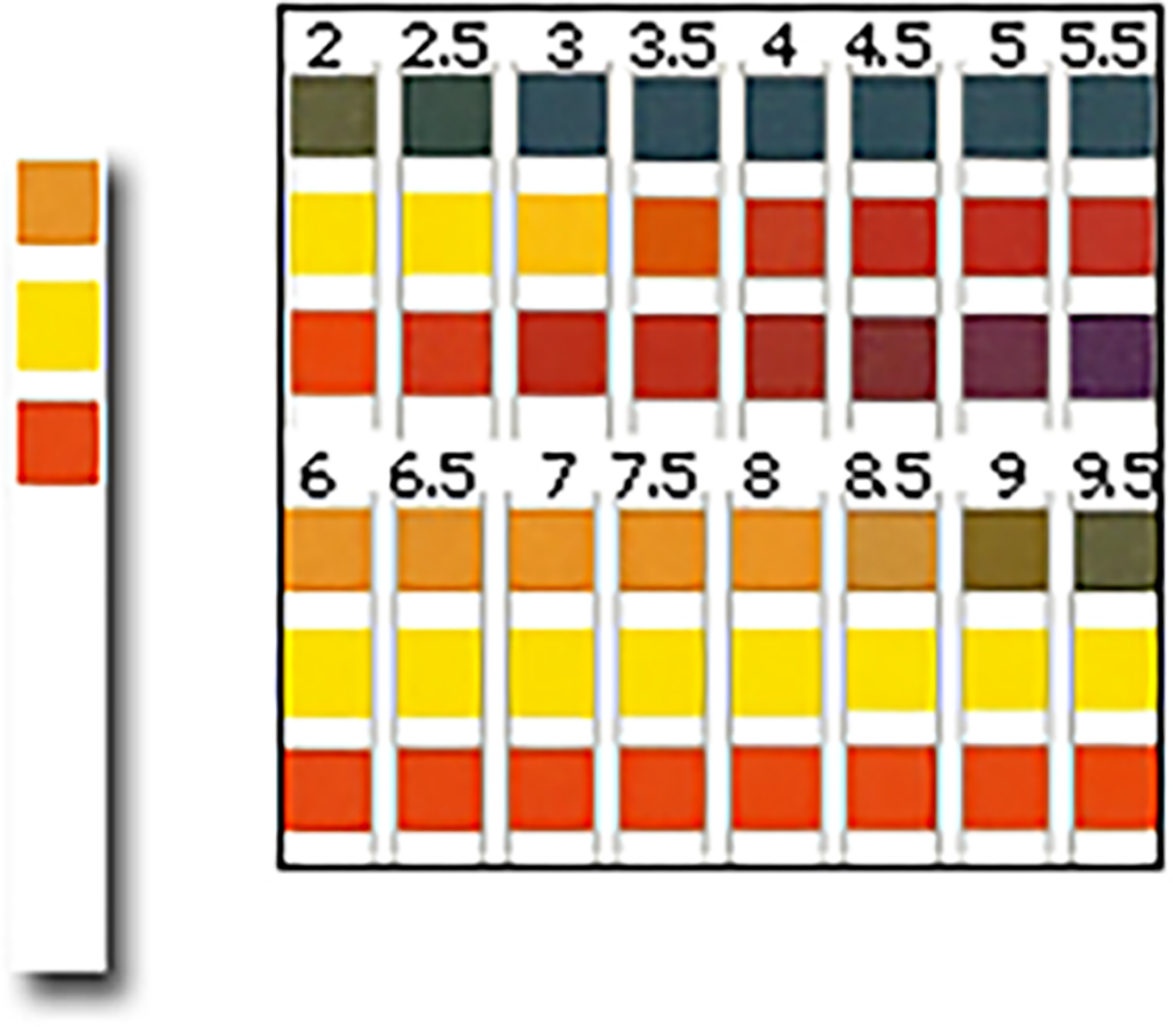
Graphic representation of a generic pH strips and colour scale for NG-tube placement.

An online simulation of pH strips was used to test both a group of health care professionals and a group of novices to perform a pH level discrimination test (DT). Only professionals were also invited to fill TAM, TIU. The DT was developed to show participants ten pH strips (available on the market) with a three-colour pad indicator (see Fig 2).

Five of the test strips were generated by a direct copy and paste from the colour scale for a commercially available strip that indicate varying levels of pH. Thus these five stimuli represented the perfect visibility condition (i.e. were the Control Stimuli).

The other five were pictures of real impregnated strips; those represented a more realistic condition of strip visibility (Target Stimuli). Each set of Target and Control stimuli contained strips with pH from 4 to 6, with 0.5 intervals. Each strip was displayed online at 350dpi to ensure a high quality of the image, with a natural dimension of 90 x 40mm. Strips were randomly presented to participants who were invited to match the pH level represented by each one of the ten stimuli with a colour scale (pH from 2 to 9) and to report the correct pH value.

An initial trial of the DT was performed with a sample of five professionals, including nurse, doctors and experts of human factors to check the clarity of the instructions and questions, and the average time to perform the DT. Amendments were implemented by three of the authors SB, PB and ZA.

The aim of the DT was to identify, in a simulated and stress-free situation the likelihood of errors in reading commercially available pH strips.

Two hypotheses about performance of DT participants were proposed as follows:

Hp1 The readability condition makes a significant difference in the correct discrimination of the strips. Strips generated for the control stimuli that exactly match the colour scale will have significantly less reading errors than the target stimuli (i.e. real impregnated strips.)
Hp2 Experience in the use pH strips will significantly improve the ability to perform correct discrimination of pH strips. Professionals are used to performing pH strip reading thus there is expected to be a difference between the professionals’ performance on the discrimination tests (i.e. the control and target) stimuli when compared to novices, with professionals making significantly fewer errors.

## Results

### Review of practice

The audit of current practice included 24 patients. Patients received a total of 37 procedures of NG-tube insertions–the average number of procedures per patient was 1.5 (range 1–4 insertions). In 45.7% of the cases aspiration cannot be achieved and 54% of the NG-tube insertions required x-ray confirmation.

Our analysis also found that the failure to aspirate fluid is significantly lower (29.2%) at the time of initial NG-tube insertion. Importantly, if fresh NG-tubes have to be inserted (and a re-test is required) then it becomes less likely that the aspiration of gastric contents is successful.

After a pH strip confirmation of tube position, the patient waited an average of 4 hours and 30 minutes before feeding and medication started. After an x-ray, the patient waiting time increased to an average of 17 hours and 18 minutes.

### Semi-structured interview

Participants were NHS nurses (n = 20) with at least three years of experience in the use of pH strips. One participant was excluded from the analysis, because s/he failed to complete all the steps required (see Table 1). All participants stated that they had never received training in the use of pH strips to test human gastric aspirate. However, the 78.9% of participants received formal or informal training in the use of hydrophilic NG-tube for enteral feeding.

**Table 1:** Demographic data of interviewees.

**Gender**	**n**	**%**	**Place of work**	**n**	**%**
Male	4	21.1%	Secondary	15	78.9%
Female	15	78.9%	Tertiary	4	21.1%
**Training with hydrophilic** **NG-Tube**	**n**	**%**	**Training with pH strips for** **NG-tube**	**n**	**%**
None	4	21.1%	None	19	100%
Informal	2	10.5%			
Formal	13	68.4%			
**Age**	**n**	**%**	**Experience with pH strips**	**n**	**%**
18–24 Years old	1	5.2%	From more than 3 to 10 years	11	58.9%
25–35 Years old	4	21.1%	More 10 years	8	42.1%
36–45 Years old	9	47.4%			
> 46	5	26.3%			

**Fig 3:**
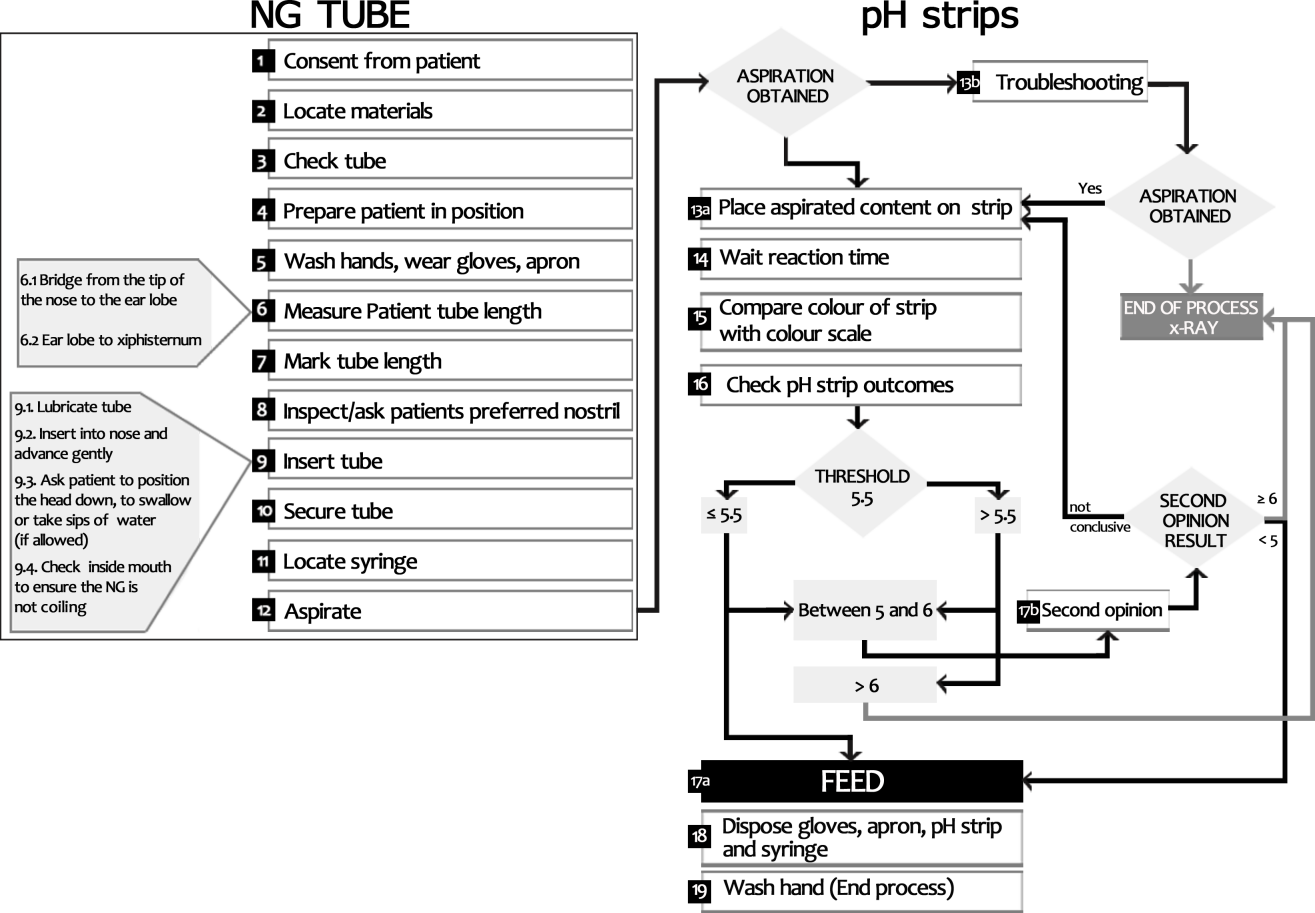
Co-designed process of use of hydrophilic NG-tube and pH strips to check the tube position.

At the beginning of the interview the graphic representation of NG-tube and pH strips use (S2 Fig) was used to enable participants to comment, revise and assist in the co-design of the process of use. All feedback was incorporated and used to develop the final representation in Fig 3. Three key points emerged from the interview:

- 36.8% of professionals reported that it would be useful to have a brochure to support the explanation and the consent phase (Fig 3, point 1). This could be used to explain to a patient why they need the procedure, and why it is important to report to a health care professional if they removed or adjusted (inadvertently or not) the NG tube.
- 68.4% of professionals stated that when the tube is inserted, in addition to pH strips confirmation, they used both visual cues and also auscultation (although these are not generally seen as reliable methods) to judge that the tube is not misplaced (Fig 3, point 9).
- 31.6% of professionals stated that they used jelly or other lubricant, instead of only water to facilitate the insertion (Fig 3, point 9.1).

The analysis of the qualitative questionnaire shows that, on the basis of their experience in the use pH strips, health care professional’s expectations are as follows:

- There is a high likelihood (64.4%) that the gastric contents cannot be aspirated, thus pH strips cannot be impregnated;
- When pH strips can be impregnated, in 66.8% of the cases pH strips fail to provide a clear outcome;
- In 35% of the cases, the correct pH strips for NG-tube check are not available in the ward, and professionals have to use a set of incorrect pH strips to perform the procedure.

These results are in line with the outcomes of the standardised questionnaires (S 4 Appendix). The overall score of UMUX-LITE [18, 19], which indicates a level of experience of usage and perceived usability of pH strips, was 47.31%. This score is equivalent to the lowest grade of usability (Grade F) on the scale [21].Despite the poor experience of use, professionals declared a small but positive level of trust (62.6%, TIU, Cronbach’s Alpha: 0.8) and acceptance (65.1%, TAM, Cronbach’s Alpha: 0.7) in the use of pH strips. Further analysis of TAM reveals that participants have a moderate level of anxiety in the use of pH strips–i.e., 49.7%. In keeping with their verbal statements, professionals declared in the TIU questionnaire that 62% of them rely on alternative methods (i.e. visual inspection, auscultation) to confirm the NG-tube position in addition to ascertaining pH. It was decided to include TAM and TIU in the online survey to better inform these findings.

### Online survey

#### Trust and acceptance

Table 2 shows the demographic characteristics of participants in the online survey (62 health professionals, 72 novices). A t-test analysis reveals that there were no differences among the scores of TIU and TAM of the health professionals who participated in the interview and those professionals who performed the survey online.

**Table 2:** Demographic characteristics of participants.

Characteristics	Health professionals		Novices	
Total numbers	62		72	
Percentage of male	19.4%		27.7%	
Percentage of female	80.6%		72.3%	
	**Mean**	**SD**	**Mean**	**SD**
Age (years)	36.9	10.0	34.9	8.5
Years of experience in using pH strips	6.2	5.2	n/a	n/a
Years of experience in healthcare	13.4	9.6	n/a	n/a

Health professionals confirm, in the online survey, that they have a positive trust (68.7%, TIU, Cronbach’s Alpha: 0.857) and acceptance (74.7%, TAM, Cronbach’s Alpha: 0.8) in the use of pH strips (S3 Appendix). The data confirms that there is a moderate level of anxiety in the use of pH strips (29.7%), and that 49.1% of professionals rely on alternative methods (i.e. visual inspection, auscultation) to confirm the NG-tube position in addition to pH determination.

#### Discrimination test

The analysis of reading the pH of the strips considered only those errors that might affect patient well-being. The following five scenarios were identified:

Secnerio 1 Feed instead of x-ray: participants reported a pH value below 5, although the value was over 6.
Secnerio 2 Retest instead of feed: participants reported a pH value between 5 or 6, although the actual value was below 5.
Secnerio 3 x-Ray instead of feed: participants reported a pH value over 6, although the actual value was below 5.
Secnerio 4 Feed instead of retest (Uncertain feed): participants reported a pH value below 5, although the displayed value was between 5 and 6.
Secnerio 5 x-Ray instead of retest: participants reported a pH value above 6, although the displayed value was between 5 and 6.

None of the participants performed Scenario 1 errors with both target and control strips, nevertheless all the other scenarios occurred in our simulation (Table 3).

**Table 3:** Scenario and percentage of errors in reading the strips during the discrimination test.

**Target**	**Percentage of errors**
Scenario 2. Retest instead of feed	3.26%
Scenario 3. x-Ray instead of feed	1.09%
Scenario 4. Feed instead of retest (Uncertain feed)	88.04%
Scenario 5. x-Ray instead of retest	7.61%
**Control**	**Percentage of errors**
Scenario 2. Retest instead of feed	38.10%
Scenario 4. Feed instead of retest (Uncertain feed)	28.57%
Scenario 5. x-Ray instead of retest	33.33%

**Fig 4:**
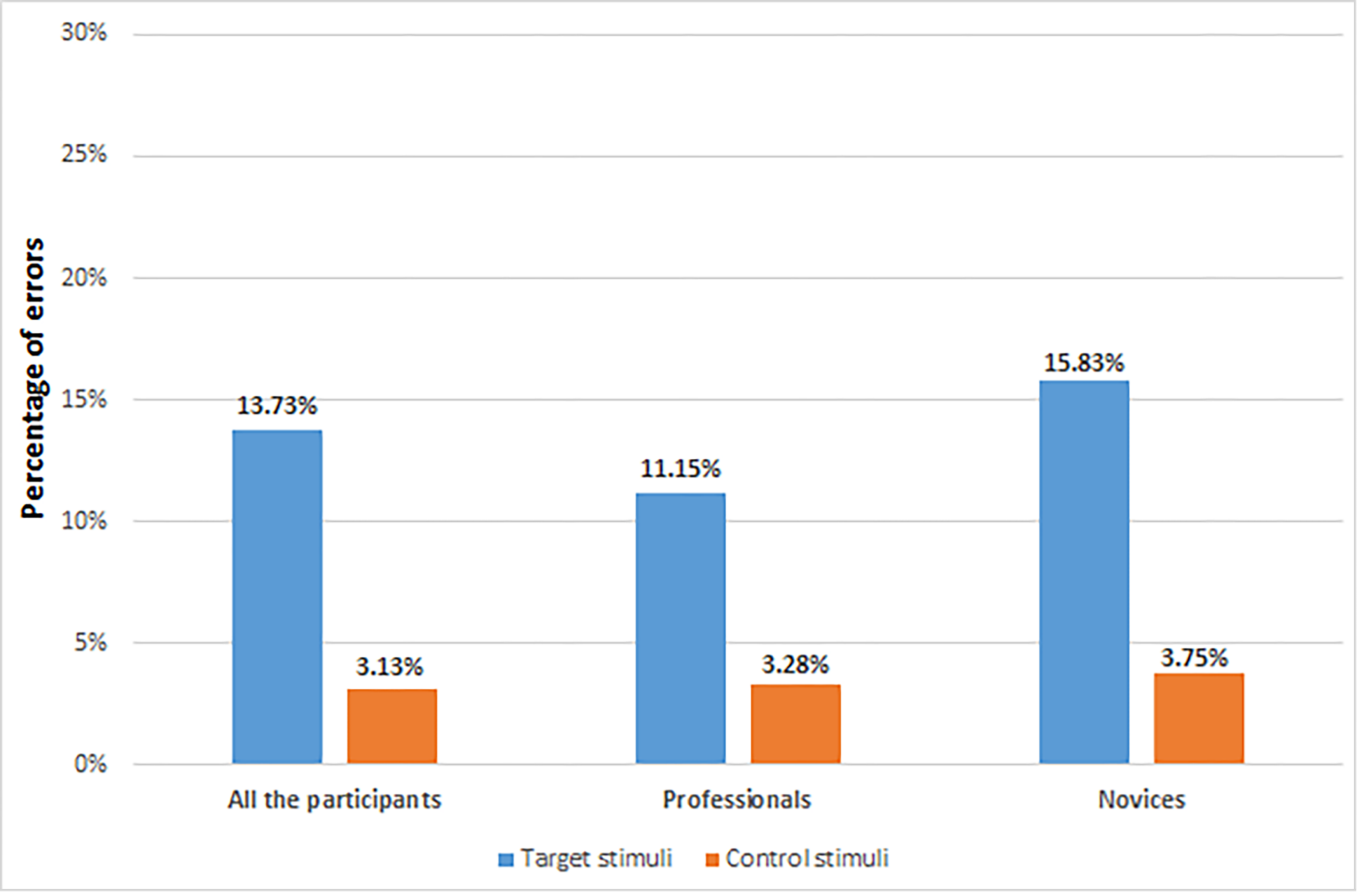
Percentage of errors in the discrimination test.

Fig 4 shows the percentage of errors made by professionals and novices in reading Control and Target strips.

In line with our expectations (Hp1), both the health professionals and the novices made significantly less errors (t (133), p < .001) in reading and correctly recognising the ‘Control’ stimuli (M: .15; SD: .07) then the ‘Target’ stimuli (M: .68, SD: .66).

Contrary to the expectations (Hp2), there was no significant difference between professionals and novices in the number of errors in reading and reporting the value of both Control and Target stimuli.

## Discussion

Findings of present study outlined at least three main issues in the use of use of pH strips for confirmation of NG-tube position that can be listed as follows:

There is a misperception amongst health care professionals regarding the feasibility of using pH strips to confirm the location of nasogastric tubes. They perceived that obtaining an aspirated sample is even harder (64.4%) compared to our findings in real clinical settings (45.7%) and those reported from other studies [2, 9]. This mismatch between perceived issues in performing the procedure and the clinical reality is more concerning if we consider that the likelihood of failing to obtain an aspirated sample reduces to 29.2% when the patient first receives the NG-tube procedure. This mismatch could explain why an x-Ray is still used more often than the pH strip as a placement confirmation check, even though our data suggest the use of an x-Ray delays patient feeding (17 hours and 18 minutes) compare to the pH approach (4 hours and 30 minutes).

The complexity of the process of pH strip confirmation is underestimated by professionals and hospitals administration. As Fig 3 shows the process of use is composed of at least 19 steps, with several decision-making points. To handle these steps in a complex and stressful environment, such as a busy hospital ward, professionals have to be adequately trained. Nevertheless, our data suggested that none of the nurses we interviewed had been trained in the use and management of pH strips. However, many had lengthy experience in using the strips for the procedure, and 84.2% of them declared that they received training in using strips for other procedures (e.g. urine test). All those interviewed who had received formal or informal training had stated that they were instructed to only use sterile water to lubricate hydrophilic NG-tube as this type of tube can be obstructed by other types of lubricants. Despite this 31.5% of nurse stated that they quite often used a lubricant instead of sterile water during the procedure to facilitate the hydrophilic NG-tube insertion, although they were aware that this could compromise the use of the pH strips. It would appear that they chose to concentrate their attention on how to manage and place the tube, with less emphasis being placed on the management and use of pH strips. This low level of attention to pH strips may also explain why 35% of professionals have stated that they used an incorrect set of pH strips on occasions.

The overall experience of usage of pH strips among professionals is poor. From the professionals’ point of view, the pH strips are not reliable, and often professionals could not obtain a clear (66.8%) or readable result (43.4%). The perceived usability of strips is 47.3% (i.e., well below an acceptable level.) Despite this, the healthcare professionals declared a positive level of trust (ranging from 62.6%, to 68.7%) and acceptance (ranging from 65.1% to 74.7%), although they also claimed to have a quite high level of anxiety in the use of strips (ranging from 29.7% to 49.7%).

Our simulation (DT) shows that there are no differences in error making by professionals and novices when reading the strips. The acquisition of experience in the clinical setting with pH strips is not enough to proficiently use this method, suggesting poor strip design. Our data suggested that professionals in the simulated condition have misinterpreted 11 out of 100 (target) strips. This represents an error rate of 11.15% (Fig 2). These findings are in line with others reports, in which clinicians misinterpreted 12 out of 100 strips using similar criteria to those in current study [11].

The likelihood of misreading could further increase due to performance influencing factors such as time constraints, stress and inappropriate lighting conditions. Other research[24] has demonstrated discrepancies between pH meter reading and pH paper reading. This further questions the reliability of methods to ascertain pH, and highlights the need for improved methods to ascertain the position of NG feeding tubes.

In our study, professionals used their experience and knowledge to find strategies to minimise the potential for error. 68.4% of nurses stated that they are used to combining the pH strip reading with other methods. The use of alternative methods was confirmed in the ‘trust’ questionnaire by both the interviewed (62%) and the surveyed professionals (49.1%).

Nurses stated that they use visual inspection of signs and symptoms of respiratory distress, visual analysis of gastric contents and in some cases auscultation in association with pH strips to reduce their uncertainty. This is in keeping with other research finding [25–27]. Although this empirical approach of combining methods could expose patient to risks, it is also a sign of the resilience [28] of healthcare professionals who, when working in settings where a product is perceived as unsafe and unusable, try to reduce the uncertainties and increase the safety for patients. Moreover this approach of combining different methods to check NG-tube position was recently supported by a study [29] that also argues for further research on a combination of methods to improve the accuracy of confirmation of NG-tube position.

## Conclusion

We have answered the four questions set in this study as follows:

1. Is the use of pH strips perceived as simple and safe by clinicians?
  Professionals underestimate the complexity of using pH strips to ascertain the position of the NG-tube. The process of positioning and checking the tube using pH strips is actually a multi-task process with various points requiring decision making. The usability and experience of the use of pH strips among professionals is poor.
2. What is the likelihood of misreading that could potentially affect the clinicians’ decision making?
  The likelihood of misreading the strips that may lead to errors of decision making with an adverse impact on a patient is equal to 11.15%.
3. What are the cause of errors?
  The lack of usability of pH strips, and the lack of formal training in performing the multi-tasks required for decision making may be causes of error. The finding that there was no difference between the errors made by both clinicians and non-clinicians in reading the strips suggest a need of pH strip redesign.
4. What are the potential mitigation strategies?

Four mitigation strategies can be outline in tune with our results

- The first, and most important mitigation strategy is a redesign of pH strips. pH strips originally designed for laboratory use are today commonly used in the field. Our results indicate a need to design pH strips specifically for bed-side use. These would need to be both easy to learn and use. These may, for example, be able to provide a simple ‘yes’ or ‘no’ answer to the question “is the NG-tube in the stomach?” To achieve such a goal, for instance, innovative approaches such as the development of a test to detect lipase^6^ could be further explore.
- Our results have also important implications for healthcare professionals. Hospital administrators need to consider whether training strategies for the use of pH strips to check NG-tube position are available and effective. In this study, many professionals had not been trained in the use of pH strips nor were they aware of the potential errors in decision making due to misreading. Training is known to be effective in reducing errors in the misinterpretation of radiography by doctors from 4% to 0.05% [29] and lessons may be learned from this and other applications.
- Regulators may need to rethink guidelines to better support professionals. Regulators should investigate the possibility of reducing the pH threshold for feeding from ≤5.5 to ≤5.0, as this is already shown to be a way to reduce failures in detecting misplacement [30–32].
- Finally the research community might consider [29] the development of further tests and standardised low-cost bedside methods which could be combined with pH strips to reduce uncertainty. The development and the validation of combined methodologies could also avoid those empirical strategies, based solely on professional experience, that are found in current practice.

Our findings strongly resonate with, and attempt to extend results of, previous studies to propose operative solutions for checking the position of a NG-tube with pH strips. However, the present study gathered data mainly throughout a combination of qualitative methods and simulation with a limited sample of participants. This limitation could be accounted for in future studies by undertaking larger observational and clinical studies to test our findings and conclusions and to evaluate the usefulness of the proposed mitigation strategies.

## Supporting information

S1 Fig Preliminary graphic representation of NG-tube and pH strips use. (TIF)

S2 Fig Revised graphic representation of NG-tube and pH strips use. (TIF)

S1 Appendix Questionnaires and evaluation scales. (DOCX)

S2 Appendix Semi-structured interview—Results of standardised questionnaire. (DOCX)

S3 Appendix Online survey—Results of standardised questionnaire. (DOCX)
