# Supplementary figures and images for: Usability study of pH strips for nasogastric tube placement
Source: PLoS One. 2017 Nov 30;12(11):e0189013. doi: 10.1371/journal.pone.0189013 (PMC5708821; doi:10.1371/journal.pone.0189013)

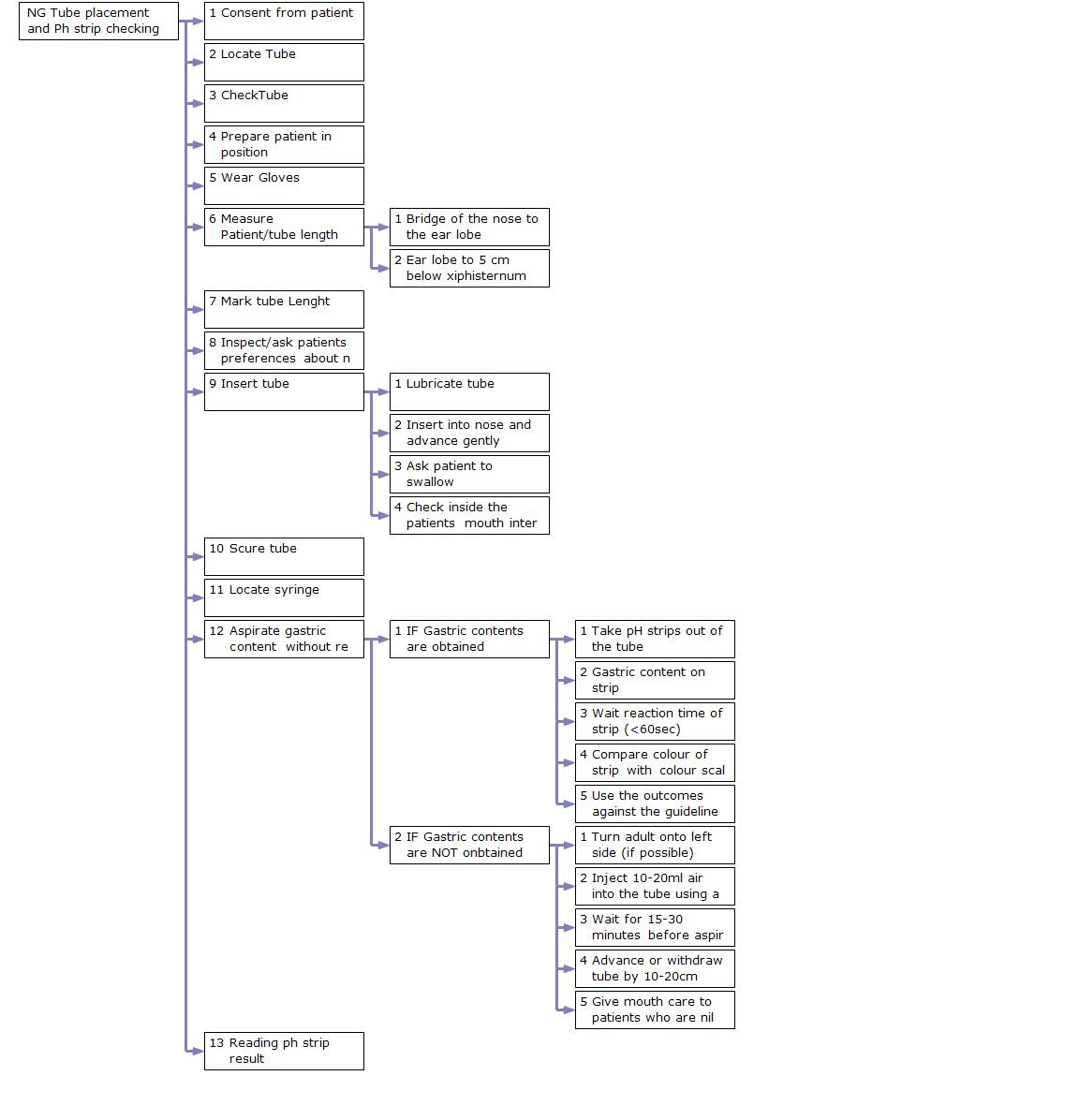

Supplement: S1 Fig — (TIF) [file pone.0189013.s001.tif]

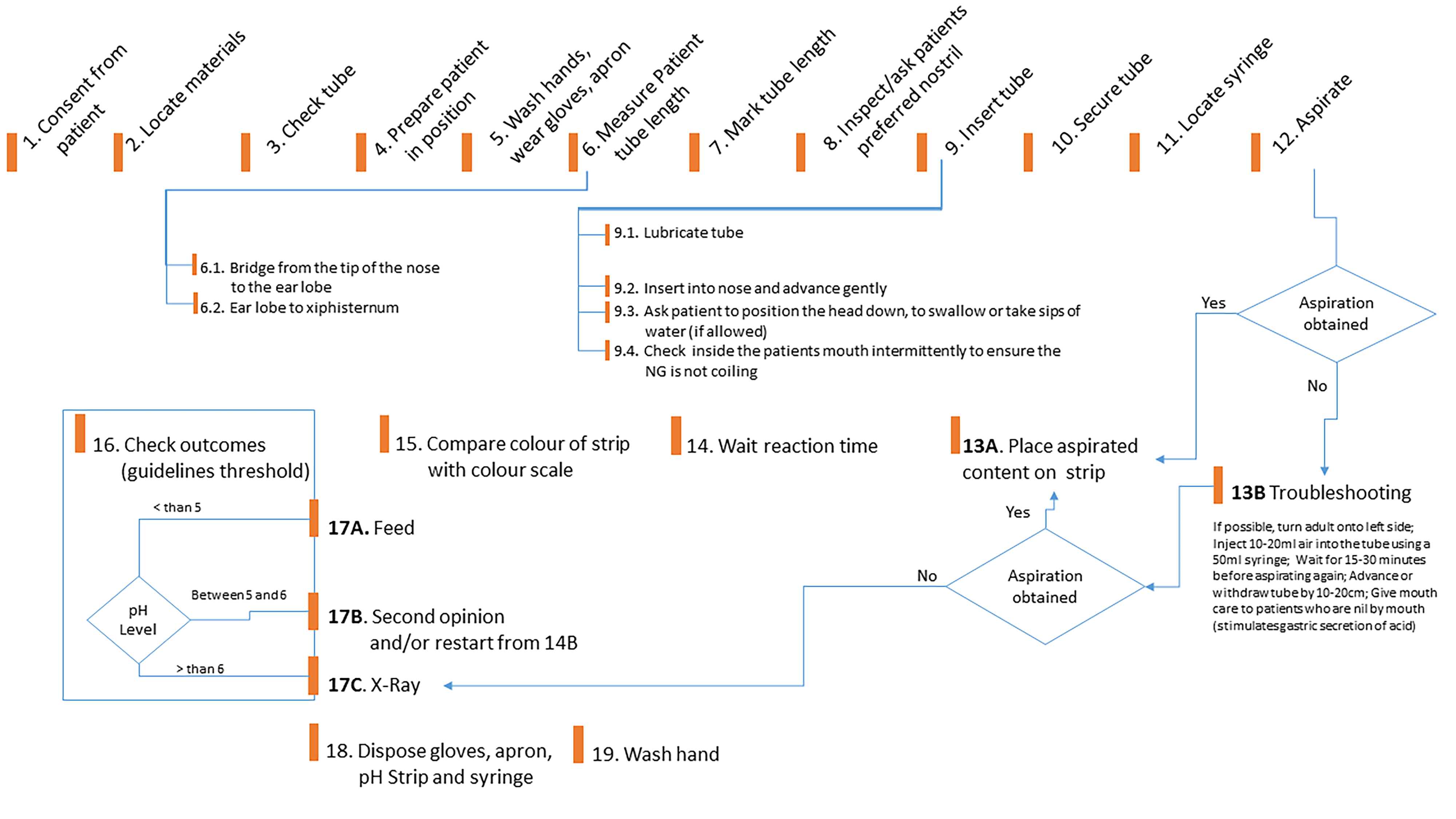

Supplement: S2 Fig — (TIF) [file pone.0189013.s002.tif]
