# Supplementary material for: Usability study of pH strips for nasogastric tube placement
Source: PLoS One. 2017 Nov 30;12(11):e0189013. doi: 10.1371/journal.pone.0189013 (PMC5708821; doi:10.1371/journal.pone.0189013)
Supplement: S1 Appendix — (DOCX) [file pone.0189013.s003.docx]

**S1 Appendix. Evaluation scales - demographic, experience in the last 12 months, trust in use, acceptance and perceived usability.**

**Section A. Demographic and qualitative questionnaire**

Demographic - Information About You

We would like to collect some information about you, and your expertise in the use of pH Strips to checking the position of the nasogastric feeding tube.

| 1. Your place of work… *(please, mark all that apply)*  - Primary care - GP surgery - Primary care - Social services - Secondary care - NHS Hospital Trust - Secondary care – other, please specify: ________________________ - Tertiary care |
| --- |
| 1. Level of experience… *(please, mark all that apply)*  - Doctor - Nurse - Paramedic - Pharmacist - Physiotherapist - Dietician - Other, please specify: _______________________________ |
| 1. Your gender is  - Male - Female - I prefer not to say |
| 1. Are you used to checking the position of the NG tube with pH strips? *(please, mark only one box)*  - Not at all. - Yes, but only when I have to. - Yes, on regular basis. |
| 1. Do you suffer from colour vision deficiency (colour blindness)? *(please, mark only one box)*  - No - Yes |
| 1. Have you received training in the use of pH strips and nasogastric feeding tubes?  - Not at all. - Yes, I received informal training at _____________________Year: - Yes, I received formal training at _______________________Year: |
| 1. How many years of experience do you have using pH strips to check NG tube positioning? _______ |
| 1. Your Age:   _____ |

Experience in the use of pH strips to ascertain a NG tube position in the last 12 months

**Instructions**: For each of the following statements, mark your agreement with an X from 0 (Never) to 100% (always)

Example:


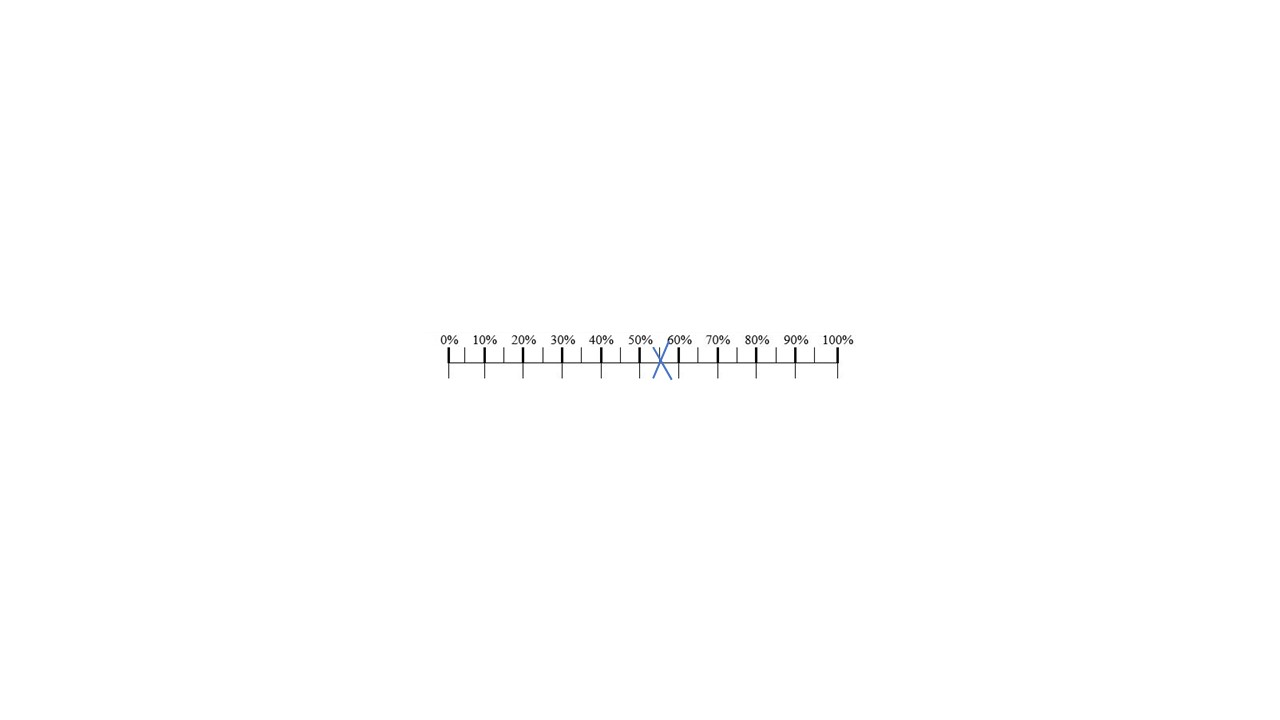


| In the last 12 months… | | |  |
| --- | --- | --- | --- |
| 1 | …I was able to obtain an aspiration to impregnate the strips | |  |
|  |  | \| 0% \| \| 10% \| \| 20% \| \| 30% \| \| 40% \| \| 50% \| \| 60% \| \| 70% \| \| 80% \| \| 90% \| \| 100% \| \| \| --- \| --- \| --- \| --- \| --- \| --- \| --- \| --- \| --- \| --- \| --- \| --- \| --- \| --- \| --- \| --- \| --- \| --- \| --- \| --- \| --- \| --- \| \|  \|  \|  \|  \|  \|  \|  \|  \|  \|  \|  \|  \|  \|  \|  \|  \|  \|  \|  \|  \|  \|  \| \|  \|  \|  \|  \|  \|  \|  \|  \|  \|  \|  \|  \|  \|  \|  \|  \|  \|  \|  \|  \|  \|  \| | |
| 2 | …I had problems trying to correctly impregnate a pH strip | |  |
|  |  | \| 0% \| \| 10% \| \| 20% \| \| 30% \| \| 40% \| \| 50% \| \| 60% \| \| 70% \| \| 80% \| \| 90% \| \| 100% \| \| \| --- \| --- \| --- \| --- \| --- \| --- \| --- \| --- \| --- \| --- \| --- \| --- \| --- \| --- \| --- \| --- \| --- \| --- \| --- \| --- \| --- \| --- \| \|  \|  \|  \|  \|  \|  \|  \|  \|  \|  \|  \|  \|  \|  \|  \|  \|  \|  \|  \|  \|  \|  \| \|  \|  \|  \|  \|  \|  \|  \|  \|  \|  \|  \|  \|  \|  \|  \|  \|  \|  \|  \|  \|  \|  \| | |
| 3 | …I had problems in reading the pH strips easily and correctly | |  |
|  |  | \| 0% \| \| 10% \| \| 20% \| \| 30% \| \| 40% \| \| 50% \| \| 60% \| \| 70% \| \| 80% \| \| 90% \| \| 100% \| \| \| --- \| --- \| --- \| --- \| --- \| --- \| --- \| --- \| --- \| --- \| --- \| --- \| --- \| --- \| --- \| --- \| --- \| --- \| --- \| --- \| --- \| --- \| \|  \|  \|  \|  \|  \|  \|  \|  \|  \|  \|  \|  \|  \|  \|  \|  \|  \|  \|  \|  \|  \|  \| \|  \|  \|  \|  \|  \|  \|  \|  \|  \|  \|  \|  \|  \|  \|  \|  \|  \|  \|  \|  \|  \|  \| | |
| 4 | …I had problems in obtaining reliable results using pH strips | |  |
|  |  | \| 0% \| \| 10% \| \| 20% \| \| 30% \| \| 40% \| \| 50% \| \| 60% \| \| 70% \| \| 80% \| \| 90% \| \| 100% \| \| \| --- \| --- \| --- \| --- \| --- \| --- \| --- \| --- \| --- \| --- \| --- \| --- \| --- \| --- \| --- \| --- \| --- \| --- \| --- \| --- \| --- \| --- \| \|  \|  \|  \|  \|  \|  \|  \|  \|  \|  \|  \|  \|  \|  \|  \|  \|  \|  \|  \|  \|  \|  \| \|  \|  \|  \|  \|  \|  \|  \|  \|  \|  \|  \|  \|  \|  \|  \|  \|  \|  \|  \|  \|  \|  \| | |
| 5 | …I had more problems than answers - using pH strips | |  |
|  |  | \| 0% \| \| 10% \| \| 20% \| \| 30% \| \| 40% \| \| 50% \| \| 60% \| \| 70% \| \| 80% \| \| 90% \| \| 100% \| \| \| --- \| --- \| --- \| --- \| --- \| --- \| --- \| --- \| --- \| --- \| --- \| --- \| --- \| --- \| --- \| --- \| --- \| --- \| --- \| --- \| --- \| --- \| \|  \|  \|  \|  \|  \|  \|  \|  \|  \|  \|  \|  \|  \|  \|  \|  \|  \|  \|  \|  \|  \|  \| \|  \|  \|  \|  \|  \|  \|  \|  \|  \|  \|  \|  \|  \|  \|  \|  \|  \|  \|  \|  \|  \|  \| | |
| 6 | …I found my liability may be compromise by only ascertaining NG tube position through pH strips | |  |
|  |  | \| 0% \| \| 10% \| \| 20% \| \| 30% \| \| 40% \| \| 50% \| \| 60% \| \| 70% \| \| 80% \| \| 90% \| \| 100% \| \| \| --- \| --- \| --- \| --- \| --- \| --- \| --- \| --- \| --- \| --- \| --- \| --- \| --- \| --- \| --- \| --- \| --- \| --- \| --- \| --- \| --- \| --- \| \|  \|  \|  \|  \|  \|  \|  \|  \|  \|  \|  \|  \|  \|  \|  \|  \|  \|  \|  \|  \|  \|  \| \|  \|  \|  \|  \|  \|  \|  \|  \|  \|  \|  \|  \|  \|  \|  \|  \|  \|  \|  \|  \|  \|  \| | |
| 7 | …I was forced to use not CE market pH strips (not for in-vitro use) to ascertain NG tube position | |  |
|  |  | \| 0% \| \| 10% \| \| 20% \| \| 30% \| \| 40% \| \| 50% \| \| 60% \| \| 70% \| \| 80% \| \| 90% \| \| 100% \| \| \| --- \| --- \| --- \| --- \| --- \| --- \| --- \| --- \| --- \| --- \| --- \| --- \| --- \| --- \| --- \| --- \| --- \| --- \| --- \| --- \| --- \| --- \| \|  \|  \|  \|  \|  \|  \|  \|  \|  \|  \|  \|  \|  \|  \|  \|  \|  \|  \|  \|  \|  \|  \| \|  \|  \|  \|  \|  \|  \|  \|  \|  \|  \|  \|  \|  \|  \|  \|  \|  \|  \|  \|  \|  \|  \| |  |
| 8 | …I ascertained NG tube position with pH strips with a graduation/calibration of 1 instead of 0.5 | |  |
|  |  | \| 0% \| \| 10% \| \| 20% \| \| 30% \| \| 40% \| \| 50% \| \| 60% \| \| 70% \| \| 80% \| \| 90% \| \| 100% \| \| \| --- \| --- \| --- \| --- \| --- \| --- \| --- \| --- \| --- \| --- \| --- \| --- \| --- \| --- \| --- \| --- \| --- \| --- \| --- \| --- \| --- \| --- \| \|  \|  \|  \|  \|  \|  \|  \|  \|  \|  \|  \|  \|  \|  \|  \|  \|  \|  \|  \|  \|  \|  \| \|  \|  \|  \|  \|  \|  \|  \|  \|  \|  \|  \|  \|  \|  \|  \|  \|  \|  \|  \|  \|  \|  \| |  |
| 9 | …With a new patient (first positioning) I requested a x-Ray because the pH strip outcomes were uncertain | |  |
|  |  | \| 0% \| \| 10% \| \| 20% \| \| 30% \| \| 40% \| \| 50% \| \| 60% \| \| 70% \| \| 80% \| \| 90% \| \| 100% \| \| \| --- \| --- \| --- \| --- \| --- \| --- \| --- \| --- \| --- \| --- \| --- \| --- \| --- \| --- \| --- \| --- \| --- \| --- \| --- \| --- \| --- \| --- \| \|  \|  \|  \|  \|  \|  \|  \|  \|  \|  \|  \|  \|  \|  \|  \|  \|  \|  \|  \|  \|  \|  \| \|  \|  \|  \|  \|  \|  \|  \|  \|  \|  \|  \|  \|  \|  \|  \|  \|  \|  \|  \|  \|  \|  \| |  |
| 10 | …With a patient with tube already in place, I requested a x-Ray because the pH strip outcomes were uncertain | |  |
|  |  | \| 0% \| \| 10% \| \| 20% \| \| 30% \| \| 40% \| \| 50% \| \| 60% \| \| 70% \| \| 80% \| \| 90% \| \| 100% \| \| \| --- \| --- \| --- \| --- \| --- \| --- \| --- \| --- \| --- \| --- \| --- \| --- \| --- \| --- \| --- \| --- \| --- \| --- \| --- \| --- \| --- \| --- \| \|  \|  \|  \|  \|  \|  \|  \|  \|  \|  \|  \|  \|  \|  \|  \|  \|  \|  \|  \|  \|  \|  \| \|  \|  \|  \|  \|  \|  \|  \|  \|  \|  \|  \|  \|  \|  \|  \|  \|  \|  \|  \|  \|  \|  \| |  |

**Section B. standardised questionnaire**

Acceptance

This section contains 13 statements to assess general acceptance of PH strips tools to rapidly inform your decision making about nasogastric tube positioning.

*Instructions*: For each of the following statements, mark one box that best describes the use of pH strips in your practice from **1 (strongly disagree) to 7 (strongly agree)** *

| 1. I would find pH strips useful in my job. | 1 | 2 | 3 | 4 | 5 | 6 | 7 |
| --- | --- | --- | --- | --- | --- | --- | --- |
| 1. Using pH strips enables me to make decisions more quickly. | 1 | 2 | 3 | 4 | 5 | 6 | 7 |
| 1. Using pH strips for clinical decision making is a good idea. | 1 | 2 | 3 | 4 | 5 | 6 | 7 |
| 1. I like working with pH strips. | 1 | 2 | 3 | 4 | 5 | 6 | 7 |
| 1. Those people who influence my behaviour think that I should use pH strips. | 1 | 2 | 3 | 4 | 5 | 6 | 7 |
| 1. In general, both hospital management and colleagues have supported the use of pH strips. | 1 | 2 | 3 | 4 | 5 | 6 | 7 |
| 1. I have the knowledge necessary to use pH strips. | 1 | 2 | 3 | 4 | 5 | 6 | 7 |
| 1. I could usually complete a clinical task using pH strips even if there was no one around to tell me what to do. | 1 | 2 | 3 | 4 | 5 | 6 | 7 |
| 1. I could usually complete a clinical task using pH strips if I could call someone for help if I got stuck. | 1 | 2 | 3 | 4 | 5 | 6 | 7 |
| 1. I feel apprehensive about using pH strips. | 1 | 2 | 3 | 4 | 5 | 6 | 7 |
| 1. I hesitate to use pH strips for fear of decision-making mistakes. | 1 | 2 | 3 | 4 | 5 | 6 | 7 |
| 1. pH strips are somewhat intimidating to me | 1 | 2 | 3 | 4 | 5 | 6 | 7 |
| 1. I will be using pH strips in the next 12 months. | 1 | 2 | 3 | 4 | 5 | 6 | 7 |
| 1. I have experienced problems in locating pH strips when I wanted to conduct a nasogastric aspirate test to verify tube placement | 1 | 2 | 3 | 4 | 5 | 6 | 7 |

* Statements from 1 to 13 are adapted from TAM [20]. Statement 14 was added to assess the availability in the field of pH strips to perform the procedure.

Trust

**How much trust do you have in the use of pH Strips to check the nasogastric feeding tube positioning?**

**Instructions**: For each of the following statements, mark one box that best describes your trust in the use of pH strips to check nasogastric tube position **from 1 (strongly disagree) to 7 (strongly agree)**

| - - 1. I am totally comfortable working with pH strips. | 1 | 2 | 3 | 4 | 5 | 6 | 7 |
| --- | --- | --- | --- | --- | --- | --- | --- |
| - - 1. I feel very good about how things go when I use pH strips. | 1 | 2 | 3 | 4 | 5 | 6 | 7 |
| - - 1. I always feel confident that the right things will happen when I use pH strips. | 1 | 2 | 3 | 4 | 5 | 6 | 7 |
| - - 1. It appears that things will be fine when I use pH strips. | 1 | 2 | 3 | 4 | 5 | 6 | 7 |
| - - 1. I believe that most pH strips are effective at what they are designed to do. | 1 | 2 | 3 | 4 | 5 | 6 | 7 |
| - - 1. A large majority of pH strips are excellent. | 1 | 2 | 3 | 4 | 5 | 6 | 7 |
| - - 1. I think most pH strips enable me to do what I need to do. | 1 | 2 | 3 | 4 | 5 | 6 | 7 |
| - - 1. My typical approach is to trust new pH strips that I have never used before until they prove to me that I shouldn’t trust them. | 1 | 2 | 3 | 4 | 5 | 6 | 7 |
| - - 1. I usually trust a pH strip outcome until it give me a reason not to trust it. | 1 | 2 | 3 | 4 | 5 | 6 | 7 |
| - - 1. I rely on alternative bedside methods (not x-ray) for confirmation of nasogastric tube placement in addition to aspirate pH determination | 1 | 2 | 3 | 4 | 5 | 6 | 7 |

* Statements from 1 to 9 are adapted from recent research on trust in technology [21]; statement 10 was added to assess the use of alternative methods of ascertaining NG Tube position

**Perceived user experience and usability**

The following statements have been used by others to assess the perceived experience in the use of a tools.

In this case, we will ask to you to answer the questions in relation to the use of **current** **pH strips to ascertain NG tube positioning**.

Please follow the instructions and fill in the questionnaire

**Instructions:** For each of the following statements, mark one box that best describes your overall experience with **current** **pH strips to ascertain NG tube positioning***:*

|  | Strongly Disagree |  |  |  |  |  | Strongly Agree |
| --- | --- | --- | --- | --- | --- | --- | --- |
| 1. pH strips capabilities meet my requirements | 1 | 2 | 3 | 4 | 5 | 6 | 7 |
| 1. pH strips are easy to use | 1 | 2 | 3 | 4 | 5 | 6 | 7 |

* UMUX-LITE [22-24]
