# Supplementary material for: Usability study of pH strips for nasogastric tube placement
Source: PLoS One. 2017 Nov 30;12(11):e0189013. doi: 10.1371/journal.pone.0189013 (PMC5708821; doi:10.1371/journal.pone.0189013)
Supplement: S2 Appendix — (DOCX) [file pone.0189013.s004.docx]

**S2 Appendix. Semi-structured interview: Results of standardised questionnaires.**

**Perceived user experience and usability (UMUX)**

| **Subject** | **% UMUX Overall Score** | **% Adjusted Formula** |
| --- | --- | --- |
| 1 | 40.91 | 49.49 |
| 2 | 45.45 | 52.45 |
| 3 | 27.27 | 40.63 |
| 4 | 36.36 | 46.54 |
| 5 | 40.91 | 49.49 |
| 6 | 40.91 | 49.49 |
| 7 | 54.55 | 58.35 |
| 8 | 50.00 | 55.40 |
| 9 | 36.36 | 46.54 |
| 10 | 45.45 | 52.45 |
| 11 | 36.36 | 46.54 |
| 12 | 13.64 | 31.76 |
| 13 | 36.36 | 46.54 |
| 14 | 54.55 | 58.35 |
| 15 | 36.36 | 46.54 |
| 16 | 40.91 | 49.49 |
| 17 | 31.82 | 43.58 |
| 18 | 22.73 | 37.67 |
| 19 | 22.73 | 37.67 |
| **Total** | 37.56 | **47.31** |
| *Grading scale* |  | *Grade F* |
| *Standard deviation* | *10.41* | *6.77* |
| *Cronbach’s Alpha* | *0.68* | |

**Acceptance (TAM)**

**
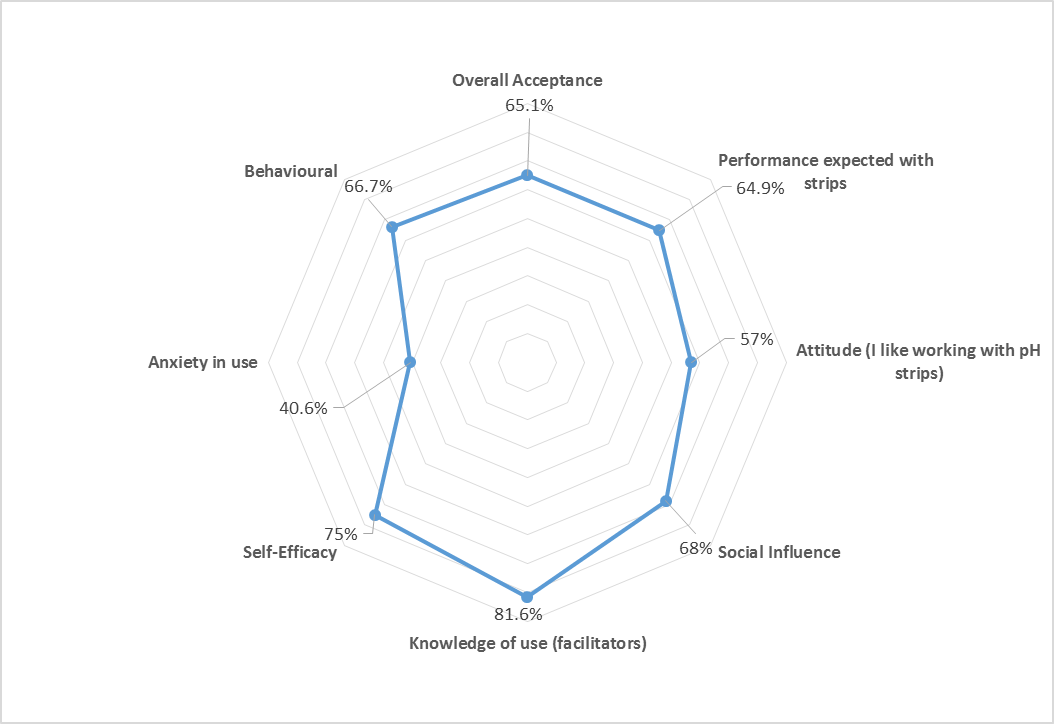
**

| **Acceptance Factors**  *Items from 1 to 13 - Appendix 2B* | **Score (%)** | **Standard deviation** |
| --- | --- | --- |
| Performance expected with strips | 64.9 | 20.4 |
| Attitude (I like working with pH strips) | 57 | 27.3 |
| Social Influence | 68 | 23.5 |
| Knowledge of use (facilitators) | 81.6 | 14.1 |
| Self-Efficacy | 75 | 17.4 |
| Anxiety in use | 40.6 | 29.9 |
| Behavioural | 66.7 | 28.5 |
| **Overall Acceptance** | **65.1** | **14.2** |
| Availability of correct pH strips in the clinical field  *Item14 - Appendix 2B* | 47.4 | 38 |

|  |  |  |
| --- | --- | --- |

**Professional trust (TIU)**


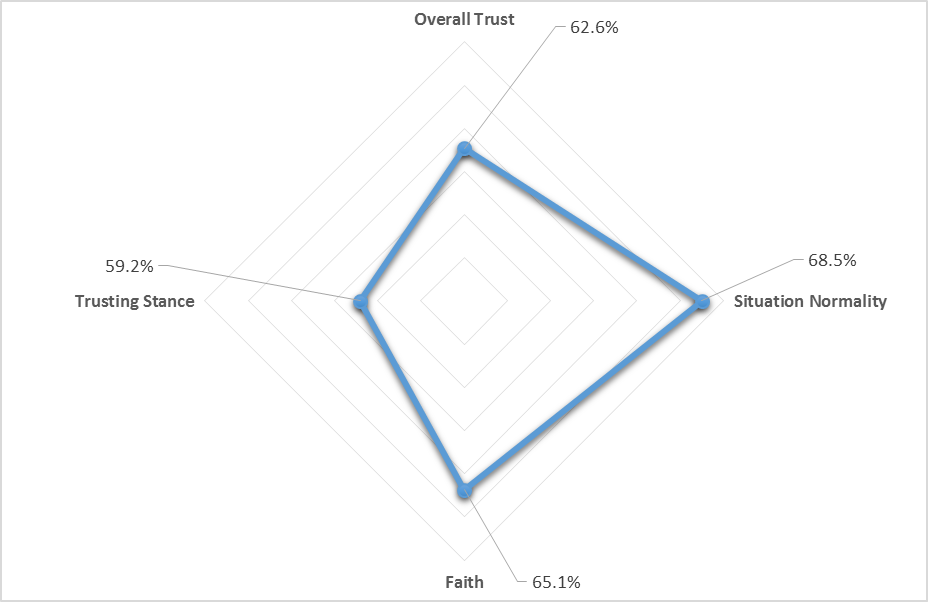


| **Trust factors**  *Items from 1 to 9 - Appendix 2B* | **Score (%)** | **Standard deviation** |
| --- | --- | --- |
| Situation Normality | 68.5 | 16.1 |
| Faith | 65.1 | 18.8 |
| Trusting Stance | 59.2 | 18.1 |
| **Overall Trust** | **62.6** | **13.9** |
| Use of alternative methods  *Item 10 - Appendix 2B* | 62 | 33.4 |
