# Supplementary material for: Usability study of pH strips for nasogastric tube placement
Source: PLoS One. 2017 Nov 30;12(11):e0189013. doi: 10.1371/journal.pone.0189013 (PMC5708821; doi:10.1371/journal.pone.0189013)
Supplement: S3 Appendix — (DOCX) [file pone.0189013.s005.docx]

**S3 Appendix. Online survey: Results of standardised questionnaires.**

**Acceptance (TAM)**

**
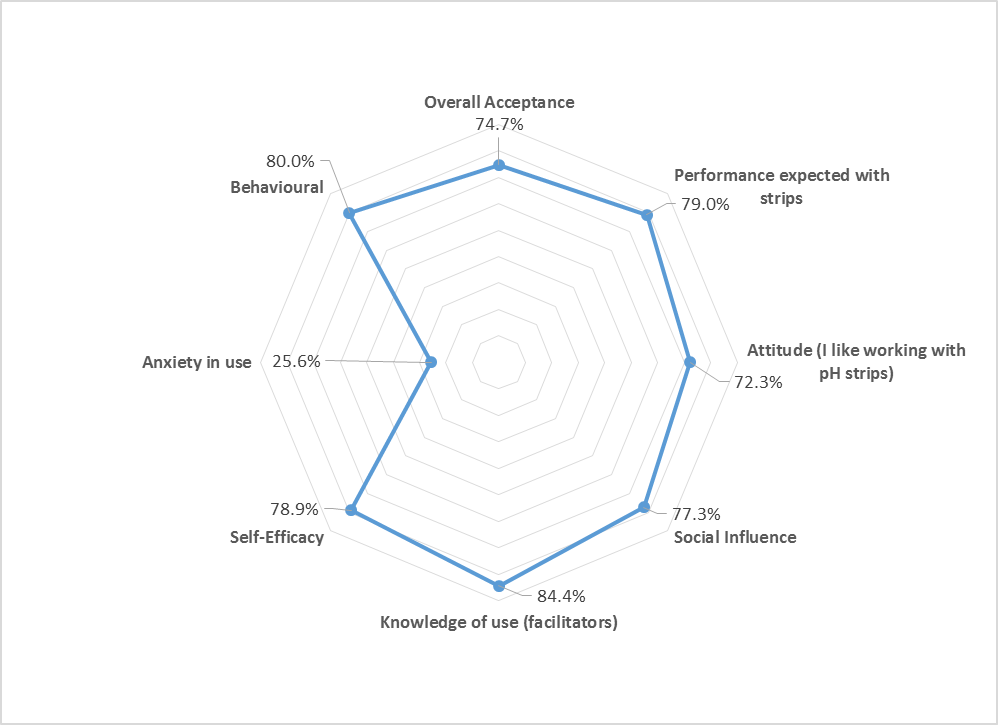
**

| **Acceptance Factors**  *Items from 1 to 13 - Appendix 2B* | **Score (%)** | **Standard deviation** |
| --- | --- | --- |
| Performance expected with strips | 79 | 20.4 |
| Attitude (I like working with pH strips) | 72.3 | 27.3 |
| Social Influence | 77.3 | 23.5 |
| Knowledge of use (facilitators) | 84.4 | 14.1 |
| Self-Efficacy | 78.9 | 17.4 |
| Anxiety in use | 29.7 | 29.9 |
| Behavioural | 80 | 28.5 |
| **Overall Acceptance** | **74.71** | **14.7** |
| Availability of correct pH strips in the clinical field  *Item14 - Appendix 2B* | 48.4 | 35 |

|  |  |  |
| --- | --- | --- |

**Professional trust (TIU)**


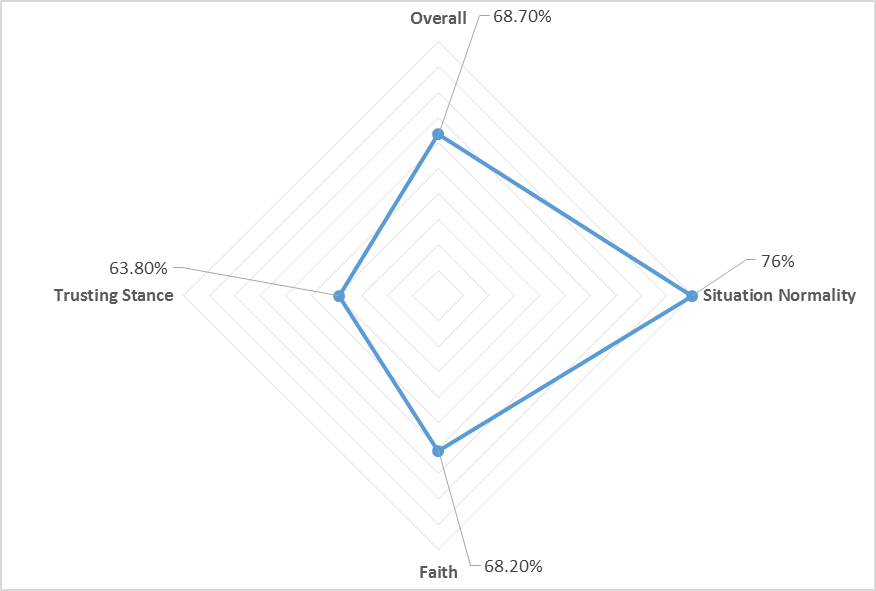


| **Trust factors**  *Items from 1 to 9 - Appendix 2B* | **Score (%)** | **Standard deviation** |
| --- | --- | --- |
| Situation Normality | 76 | 16.1 |
| Faith | 68.2 | 18.8 |
| Trusting Stance | 63.8 | 18.1 |
| **Overall Trust** | **68.7** | **13.9** |
| Use of alternative methods  *Item 10 - Appendix 2B* | 49.1 | 38 |
